# Simulation of dual-purpose chicken breeding programs implementing gene editing

**DOI:** 10.1186/s12711-023-00874-3

**Published:** 2024-01-17

**Authors:** Edward Y. S. Chuang, Robin Wellmann, Franck L. B. Meijboom, Jens Tetens, Jörn Bennewitz

**Affiliations:** 1 Institute of Animal Science, University of Hohenheim, Garbenstr. 17, 70599 Stuttgart, Germany; 2 Faculty of Veterinary Medicine, Centre for Sustainable Animal Stewardship, Utrecht University, Yalelaan 2, 3584 CJ Utrecht, The Netherlands; 3 Department of Animal Sciences, Georg-August-University Göttingen, Burckhardtweg 2, 37077 Göttingen, Germany

## Abstract

**Background:**

In spite of being controversial and raising ethical concerns, the application of gene editing is more likely to be accepted when it contributes to improving animal welfare. One of the animal welfare and ethical issues in chicken breeding is chick culling, the killing of the male layer chicks after hatching due to the poor fattening performance. Although establishing dual-purpose chicken lines could solve this problem, unfavorable genetic correlations between egg and meat production traits hindered their competitiveness. Although it is also controversial in ethical terms, gene editing may accelerate genetic progress in dual-purpose chicken and alleviate the ethical concerns from chick culling.

**Results:**

The simulation compared the utility improvement in dual-purpose use under two breeding schemes: one consisting in the improvement of the laying hens, and the second in the improvement of a synthetic line obtained from a layer broiler cross. In each breeding scheme, the breeding programs were simulated with and without gene editing. Polygenic breeding values and 500 simulated quantitative trait loci (QTL) with different levels of pleiotropy caused negative correlations between egg production, meat production, and overall health. The results of the simulation demonstrated that genetic gain could be accelerated by at most 81% for several generations if gene editing was used. The actual increase in genetic gain depended on the number of single nucleotide polymorphisms (SNPs) being edited per animal. The rate of genetic improvement became equal in scenarios with and without gene editing after 20 generations. This is because the remaining segregating QTL had small effects and their edition would have negative overall health effects from potential off-target edits. Although gene editing can improve genetic gain in quantitative traits, it can only be recommended as long as QTL with reasonable effect sizes are segregating and detectable.

**Conclusions:**

This simulation demonstrates the potential of gene editing to accelerate the simultaneous improvement of negatively correlated traits. When the risk of negative consequences from gene editing persists, the number of SNPs to be edited should be chosen carefully to obtain the optimal genetic gain.

**Supplementary Information:**

The online version contains supplementary material available at 10.1186/s12711-023-00874-3.

## Background

Gene editing (GE) is a recent technological development with a putative impact on livestock breeding schemes [1, 2]. Its application to monogenic traits for which causal variants are known and thus can be edited is straightforward, e.g., the polled locus in cattle [3, 4] or porcine respiratory and reproductive syndrome (PRRS) resistance with a defective *CD163* gene in pigs [5, 6]. Using GE for quantitative traits, however, seems to be challenging, mainly because of the difficulty to detect causal variants with a notable effect on the trait, but also because the benefit obtained from editing quantitative trait loci (QTL) with small effects might be overcompensated by the negative effects of off-target edits. Jenko et al. [7] performed a simulation to analyze the putative advantages of combining genomic selection (GS) and GE to improve quantitative traits. They called their approach promoting alleles by genome editing (PAGE) and obtained an almost double genetic gain compared to applying GS only. Although the assumptions were very optimistic [8], this study showed for the first time some prospects of GE in breeding for quantitative traits. It might be translated into practice once a powerful pipeline for the detection of causal mutations is established, which is one of the major hurdles [9].

Gene editing can be a relevant route to explore a longstanding problem in chicken breeding. Traditionally, poultry meat and egg production are based on two different and specialized crossbred lines. These are either bred for meat production (broiler line) or for egg production (layer line). As a result, the male layer line chickens cannot be used to produce meat in an economic efficient way and thus were commonly killed immediately after hatching. This ethically questionable practice evoked public discussion [10, 11] and resulted in legal bans of this practice in several countries [12]. Dual purpose lines may solve this problem, but the unfavorable genetic correlations between these two complex traits resulted in an unsatisfactory performance of dual-purpose chicken. Thus, making dual-purpose chicken competitive, is strongly desired. Gene editing may accelerate genetic progress in dual-purpose chicken, but GE also raises ethical concerns [13, 14]. Therefore, the potential use of GE to contribute to genetic progress in dual-purpose chicken is not only a technical challenge, but also comes with the need for careful ethical assessment.

Several options have been discussed to solve this problem. One option is to grow and fatten the male layer line chicken, and use them for meat production. In addition to the poor quality of their meat, this is also critically discussed from an ethical point of view, because due to the low feed and nutrient efficiency, this results in a waste of biomass that could otherwise be used much more efficiently. Furthermore, these animals need much longer fattening periods, which, assuming a constant or growing demand for chicken meat, will require much additional rearing capacity. Another option is to perform in ovo sexing and to hatch only those eggs with female chicks, which has the disadvantage that the sexing technology is hardly affordable for small-scale breeders. A third possibility is to establish a dual-purpose line with a sufficient egg and meat production level. An example is the dual-purpose Lohman dual line, which could compete with slower-growing broiler lines, but not with an intensely growing broiler line [15]. Because of the above-mentioned unfavorable genetic correlations, breeding competitive dual-purpose lines is a challenging task. The unfavorable genetic correlations are most likely due to the pleiotropic effects of causal variants. For example, Tarsani et al. [16] detected 35 single nucleotide polymorphisms (SNPs) showing antagonistic effects on egg number and body weight. Genomic selection has been proved to be a suitable tool to simultaneously improve traits with unfavorable genetic correlations and indeed has been used in breeding programs for dual-purpose chickens [17].

The aim of the present study was to explore the potential of GE in dual-purpose chicken breeding by stochastic simulations. Two alternative breeding programs were simulated. The first breeding program established a dual-purpose line from a layer line (the L-Pure), whereas the second program selected and improved a synthetic line obtained from a cross between a layer line and a broiler line (L-B cross). Both breeding programs were evaluated with and without GE.

## Methods

### Architecture of the traits

The selection index was assumed to be composed of three subindices: a subindex for meat production, a subindex for egg production, and a subindex for overall health. The three subindices were modelled as genetically correlated, quantitative traits, i.e. three traits were simulated, which reflect egg production (trait 1), meat production (trait 2), and overall health (trait 3). Dominance effects and epistatic effects were not included in the model. The phenotypic value ${y}_{ilk}$ of animal $i$ from line $l$ for trait $k$ was simulated as

$${y}_{ilk}={\mu }_{lk}+{TBV}_{ilk}+{E}_{ilk},$$

where ${\mu }_{lk}$ is the mean of trait $k$ for line $l$ in generation 0, ${TBV}_{ilk}$ is the true breeding value of animal $i$ for trait $k$, and ${E}_{ilk}$ is the normally distributed residual. It was assumed that the true breeding values can be decomposed as:

$${TBV}_{ilk}={TBV}_{ilk}^{QTL}+{TBV}_{ilk}^{poly},$$

where the term ${TBV}_{ilk}^{QTL}$ accounts for the effects of 500 simulated QTL with effect sizes exceeding a certain threshold value, and the polygenic term ${TBV}_{ilk}^{poly}$ accounts for the cumulative effect of all QTL that were not explicitly simulated because their effects were so small that they could never be detected. The QTL-based true breeding value (TBV) of trait $k$ was calculated as:

$${TBV}_{ilk}^{QTL}=\sum_{q}\left({x}_{ilq}-2{p}_{l0q}\right){a}_{qk},$$

where ${x}_{ilq}=\mathrm{0,1},2$ is the allele content of QTL $q$ in animal $i$, ${p}_{l0q}$ is the allele frequency of QTL $q$ in generation 0 of line $l$, and ${a}_{qk}$ is the additive effect of QTL $q$ for trait $k$.

### Simulation of QTL effects

The 3-vector ${\mathbf{a}}_{\mathbf{q}}$ that contains the additive effects of QTL $q$ on the three traits was sampled from a truncated multivariate t-distribution, as follows. First, the scale parameter ${\tau }_{q}^{2}$ was sampled from an inverse chi-square distribution with $\nu =4$ degrees of freedom. Then, the vector ${\mathbf{a}}_{\mathbf{q}}|{\tau }_{q}^{2}\sim {N}_{3}\left(0,{\tau }_{q}^{2}{\varvec{\Sigma}}\right)$ with additive effects was sampled conditionally on ${\tau }_{q}^{2}$ from a trivariate normal distribution with the correlation matrix ${\varvec{\Sigma}}$. The presumed correlations between egg production–meat production, egg production–overall health, and meat production–overall health were − 0.4, − 0.2, and − 0.2, respectively. If the norm $\Vert {\mathbf{a}}_{\mathbf{q}}\Vert$ of the vector was smaller than the 33%-quantile of the distribution, then the effects of this QTL were rejected and sampled again. The reason was that QTL with very small effects were covered by the polygenic part of the breeding value. Finally, the additive effects were rescaled so that the phenotypic variance of each trait was equal to 1, and the proportions of the phenotypic variances explained by the QTL were 27, 27, and 14%, respectively. The initial heritabilities were 0.4 for egg production, 0.4 for meat production, and 0.2 for overall health. The additive genetic variance of trait *k* that is explained by the QTL is denoted as ${V}_{Ak}^{QTL}$. Table 1 summarizes the variance composition of the three traits.

**Table 1:** Variance components of each trait in the founder population

Trait	Variance of QTL	Variance of polygenic effect	VE	Sum
Egg production	0.27	0.13	0.6	1.00
Meat production	0.27	0.13	0.6	1.00
Overall health	0.14	0.06	0.8	1.00

### Simulation of initial allele frequencies

The initial allele frequencies of the layer line and the broiler line were derived under the assumption that both lines originate from a common founder line that lived 100 generations in the past. The genome of the common founder line consisted of 5000 unlinked SNPs, of which 500 were QTL. The allele frequencies of the SNPs in the founder line were sampled from a beta distribution with parameters α = β = 0.5. The laying hen line and the broiler line in generation 0 differed from the founder line due to the changes in allele frequency that resulted from 100 generations of divergent selection. The expected allele frequencies in generation 0 were derived analytically. This was done by calculating for each QTL the expected change in allele frequency from one generation to the next when selection candidates are selected for breeding by truncation selection based on a selection index. The index weights used during the 100 generations of divergent selection are in Table 2, and the selection intensity was $i=1.4$. The formula for the change in allele frequency was in accordance with Table 2.2 in [18]. As the formula disregards genetic drift, the laying hen line and the broiler line in generation 0 can be interpreted as genetically diverse synthetic lines that are obtained by crossing different commercial lines. The index weights for the layer line and the broiler line were chosen such that the main production trait improved while the other traits remained approximately constant. The index weights for dual-purpose use were chosen such that the two production traits were weighted equally, while overall health remained approximately constant.

**Table 2:** Index weights for the different breeding lines

Line	Egg production	Meat production	Overall health
Layer	0.56	0.22	0.22
Broiler	0.22	0.56	0.22
Dual-purpose	0.375	0.375	0.25

### Simulation of the polygenic breeding value

The actual simulation started at generation 0 with the laying hen line and the broiler line as base populations. The 3-vector ${\mathbf{TBV}}_{\varvec{il}}^{\mathbf{poly}}$ with polygenic TBV of animal $i$ was sampled in generation 0 from a trivariate normal distribution with correlation matrix ${\varvec{\Sigma}}$. The polygenic variance ${V}_{Ak}^{poly}$ for trait $k$ in the laying hen line, the broiler line, and the cross were reduced by the same amount as the QTL-based variance ${V}_{Ak}^{poly}$ relative to the founder population. In later generations, the polygenic TBV was obtained as the sum of the parent average and the Mendelian sampling term as:

$${TBV}_{ilk}^{poly}={PA}_{ilk}^{poly}+{MT}_{ilk}^{poly},$$

where ${PA}_{ik}^{poly}$ is the parent average of individual $i$ for trait $k$. The 3-vector ${\mathbf{MT}}_{il}^{{\text{poly}}}$ with Mendelian sampling terms has the correlation matrix ${\varvec{\Sigma}}$ and satisfies:

$${MT}_{ilk}^{poly}\sim {\text{N}}\left(0,\frac{\left(1-{F}_{si}\right){V}_{Ak}^{poly}}{4}+\frac{\left(1-{F}_{di}\right){V}_{Ak}^{poly}}{4}\right),$$

where ${F}_{si}$ and ${F}_{di}$ are the inbreeding coefficients of the sire and dam, respectively. The inbreeding coefficients were calculated with R package OptiSel [19].

The actual heritabilities in generation 0 deviated from the initial trait heritabilities due to the changes in allele frequency over the 100 generations of divergent selection. The realized trait heritabilities for egg production, meat production, and overall health in generation 0 were 0.11, 0.23, and 0.10 for the laying hen line; and 0.23, 0.11, and 0.10 for the broiler line. In either line, as expected, the selection on a certain production trait decreased the heritability. The genetic correlations between the traits also changed as the result of divergent selection. In generation 0 of the laying hen line, the negative correlations of the genetic values are − 0.51 between egg production and meat production, − 0.28 between egg production and overall health, and − 0.21 between meat production and overall health. Similarly, there were negative correlations of − 0.51 between egg production and meat production, − 0.21 between egg production and overall health, and − 0.28 between meat production and overall health for the broiler line.

### Selection of the dual-purpose chicken

**Fig. 1:**
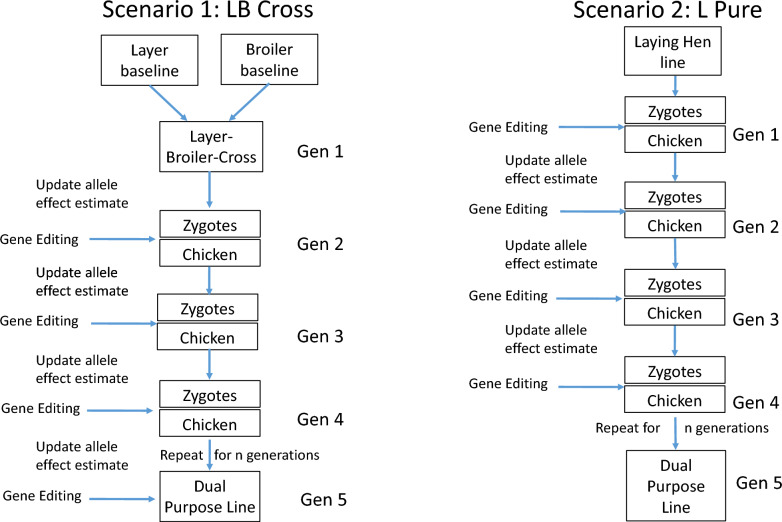
Breeding scheme for the two scenarios considered, L-B cross and L-Pure. For each scenario, breeding with and without gene editing were simulated

The breeding program for the dual-purpose chicken started at generation 0. Two alternative breeding schemes for breeding dual-purpose chicken with discrete generations were considered. In the L-Pure Scenario, a dual-purpose line was established from the laying hen base population, whereas in the L-B cross scenario, a synthetic line obtained from a cross between laying hens and broilers was selected and improved. Each line consisted of 500 animals. The mating scheme is shown in Fig. 1.

Within each line, the sires to produce the next generation were selected using optimum contribution selection (OCS) as implemented in the package optiSel [19]. The optimum contributions were calculated to maximize genetic gain in the selection index, while the rate of increase in coancestry was restricted such that an effective population size of 100 could be maintained. The coancestry was computed from pedigrees. The genetic contributions of the males were optimized, while the contributions of the females were homogeneous. That is, the contributions of the females were randomly chosen such that the numbers of offspring of different females deviated by no more than one offspring. Males and females were mated at random.

The weights of the traits in the selection index are in Table 2. The estimated breeding values (EBV) for use in OCS were simulated in accordance with [20] as:

$${EBV}_{ilk}|{TBV}_{ilk}\sim N\left({r}_{n}^{2}{TBV}_{ilk},{r}_{n}^{2}\left(1-{r}_{n}^{2}\right){V}_{Ak}\right).$$

A proof of the conditional distribution is shown in Additional file 2. The prediction reliability ${r}_{n}^{2}$ was 0.7 for the two production traits and 0.5 for overall health in generation *n* = 1. In later generations, it increased as ${r}_{n+1}^{2}={r}_{n}^{2}+0.05\left(1-{r}_{n}^{2}\right)$, which mimics the increase due to an increased reference population in genomic selection. Each scenario was replicated 100 times.

### Simulation of GE

Each breeding program was simulated with and without GE. For each breeding program, three scenarios were considered: no GE, editing five SNPs, editing 25 SNPs, and editing 100 SNPs of each top male. In the scenarios with GE, the top 25 males in each line with the highest optimum contributions were edited from generation 1 onwards. For simplicity, it was assumed that the males can be edited after their genetic value has been calculated.

The edit targets were the top SNPs with the largest estimated effects on the selection index for dual purpose production. SNPs with effects that could not be estimated because of a low minor allele frequency (MAF), and SNPs that were already in the homozygous beneficial phase in the animal were disregarded, i.e. if an SNP with a large estimated effect could not be enriched further in the animal because it already carried two beneficial alleles of that SNP, then we looked for the next-best SNP to see whether the edit was possible. This process was repeated until the desired number (5, 25 or 100) of SNPs was found. For edited SNPs, the allele count for that animal increased by 1.

In practice, a SNP might be in complete linkage disequilibrium (LD) with other SNPs, in which case an estimate ${\widehat{a}}_{nqk}$ of the SNP effect would not converge towards the true effect, but towards a suggestive effect ${a}_{{\text{qk}}}^{sugg}$. Therefore, for each SNP effect ${a}_{qk}$, a suggestive effect ${a}_{qk}^{sugg}$ was simulated by adding a normally distributed error with a variance equal to 0.2% of the additive variance. The estimate ${\widehat{a}}_{nqk}$ of the effect of SNP $q$ on trait $k$ in generation $n$ was sampled such that it converged stochastically toward the suggestive effect. A hypothetical cross of a broiler line and a laying hen line was assumed as the mapping population. SNP effects could thus be estimated even if they were not segregating anymore in the layer line. The size of the mapping population was 10,000 in the first generation, and it increased by 2000 animals with phenotypes at each generation. Hence, there were ${N}_{1}=\mathrm{10,000}$ animals in generation 1, and ${N}_{n}=2000$ additional animals in all later generations. First, for each generation $n$, a hypothetical estimate ${\widehat{a}}_{qk}^{n}$ of the SNP effect was sampled that is based only on data from a single generation. This estimate was sampled from the normal distribution ${\widehat{a}}_{qk}^{n}\sim N\left({a}_{qk}^{sugg},{\sigma }_{qkn}^{2}\right)$ with variance ${\sigma }_{qkn}^{2}=\frac{1}{2{p}_{q}\left(1-{p}_{q}\right)}\frac{{V}_{Pk}}{{N}_{n}}$, where ${p}_{q}$ is the allele frequency of QTL $q$ in the hypothetical cross, and ${V}_{Pk}\sim 1$ is the phenotypic variance of the trait. The equation for the variance ${\sigma }_{qkn}^{2}$ is derived in Additional file 2. Second, the final estimate was obtained as the pooled mean ${\widehat{a}}_{nqk}=\frac{{N}_{1}{\widehat{a}}_{qk}^{1}+\dots +{N}_{n}{\widehat{a}}_{qk}^{n}}{{N}_{1}+\dots +{N}_{n}}$. Note that the factor $\frac{1}{2{p}_{q}\left(1-{p}_{q}\right)}$ ensures that the effects of SNPs with extreme allele frequencies are estimated less accurately than the effects of SNPs with intermediate frequencies. SNPs with a minor allele frequency lower than 5% in the cross were, therefore, excluded from GE.

GE may negatively affect the overall health due to the effects of off-target edits. The negative effect on overall health was modelled by reducing the polygenic breeding value for overall health with a certain probability. The negative effect on overall health was thus transmitted by the animal to its offspring. The editing of each SNP had a 1% chance of having a negative effect on the overall health of the edited animal. The size of the negative effect was chosen as 0.05, which is close to the value of the 95% quantile of all SNP effects on overall health.

## Results

### The two lines in generation 0

Table 3 shows the mean phenotypic values of the different traits in generation 0 for the laying hen line and the broiler line. The values were obtained from 100 replicates. The traits were standardised such that they had a mean of 0 and a phenotypic variance of 1 in generation − 100. The negative genetic correlation between the traits required to give positive index weights to all traits. The overall health changed only little during the course of the breeding program, the main production trait improved by around 20 phenotypic standard deviations, and the second production trait decreased by around 2 phenotypic standard deviations.

**Table 3:** Mean and standard deviation of the phenotypes in generation 0

	Egg production	Meat production	Overall health
Layer line	19.80 (1.99)	− 2.00 (1.99)	− 0.06 (1.31)
Broiler line	− 2.14 (1.93)	19.96 (2.08)	− 0.27 (1.36)

The values were estimated from 100 replicates. Standard deviations are given in the brackets. The traits were standardised such that they had a mean of 0 and a phenotypic variance of 1 in generation − 100

Some QTL were fixed in generation 0 in their beneficial phase due to historic selection. On average, 42 QTL were fixed in the same phase in both lines, 112 additional QTL were fixed in the beneficial phase in the broiler line, and 111 QTL were fixed in the beneficial phase in the layer line. The QTL that were fixed in the same phase in both lines could not contribute to the genetic gain in the forthcoming generations.

### Genetic gain

**Fig. 2:**
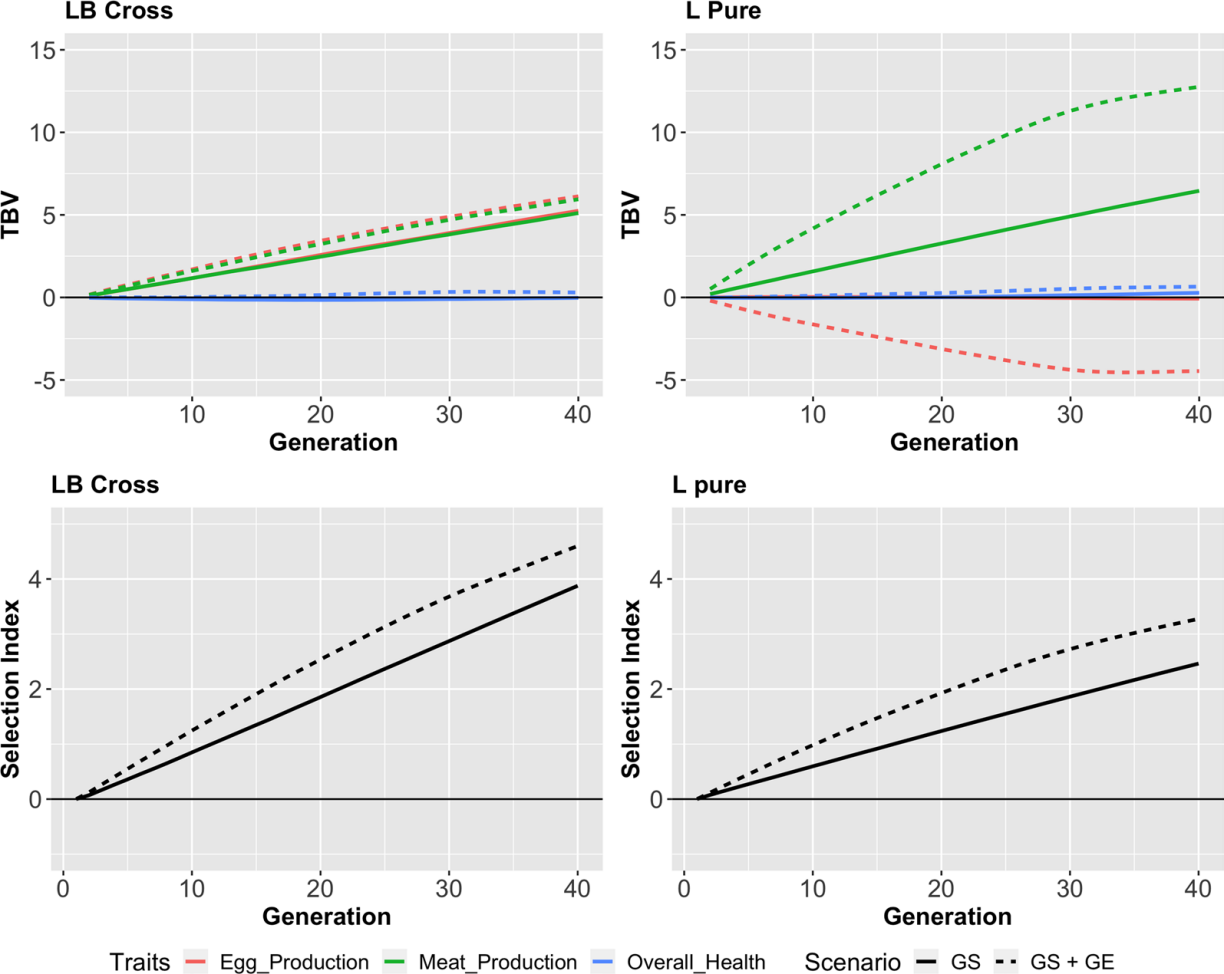
Genetic gain in the L-Pure scenario and in the L-B cross scenario without gene editing and with editing of 25 SNPs per animal. Left: The L-B cross scenario. Right: The L-Pure scenario. Top: Breeding value for all three traits. Bottom: Evaluation of the three traits with the selection index for dual-purpose production. Gene editing substantially increased meat production but reduced egg production in the L-Pure scenario. Gene editing contributed relatively more genetic gain in the L-Pure scenario than in the L-B cross scenario, but the L-B cross had an overall higher genetic gain

Figure 2 shows the genetic gain that could be achieved in a dual-purpose line with and without GE for both breeding schemes. Genetic gain in the selection index was greater in the L-B cross scenario than in the L-Pure scenario because the synthetic line that was obtained from a cross between a broiler line and a layer line had a larger genetic variance. Without GE, genetic gain in the L-Pure scenario was solely achieved by increasing meat production, whereas egg production and overall health remained constant. The numerical results are in the Additional file 1: Tables S1, S2 and S3. Gene editing enabled a faster improvement of the meat production, but, at the same time, reduced the egg production. This is because QTL with large effects on the selection index that had not yet been fixed in the layer line tended to have a large positive effect on meat production. Moreover, because of the negative genetic correlation between egg production and meat production, they also had a negative effect on egg production. This dependency is demonstrated in Table 4, which shows, for a single replicate of the L-Pure scenario, the 10 most edited QTL with their effects on egg production, meat production, overall health, and the selection index. Most SNPs have opposite effects on egg production and meat production. As shown in Fig. 2, genetic gain in the L-B cross scenario was achieved by improving both, egg production and meat production. Gene editing accelerated genetic gain only during the first generations. After generation 20, genetic gain in the scenarios with and without GE was similar.

**Table 4:** The top QTL (SNPs) being edited in a single replicate of the L-pure scenario

SNP	Number of times edited	Effect on egg production	Effect on meat production	Effect on overall health	Effect on selection index
SNP 38	453	0.0213	− 0.0186	0.0228	0.0067
SNP 22	424	0.0019	− 0.0263	0.0277	− 0.0023
SNP 161	419	− 0.0505	0.0405	− 0.0027	− 0.0031
SNP 233	397	− 0.0436	0.0469	− 0.0006	0.0011
SNP 102	370	− 0.0251	0.0093	0.0231	− 0.0002
SNP 167	367	− 0.0110	− 0.0227	0.0172	− 0.0084
SNP 20	329	− 0.0589	0.0710	0.0043	0.0056
SNP 459	327	− 0.0253	0.0188	0.0164	0.0017
SNP 174	315	0.0263	− 0.0390	0.0173	− 0.0004
SNP 376	312	0.0535	− 0.0084	− 0.0214	0.0116

The table demonstrates that the edited SNPs in the L-Pure scenario usually had opposite effects on egg production and meat production

### Scenarios with different numbers of edits

**Fig. 3:**
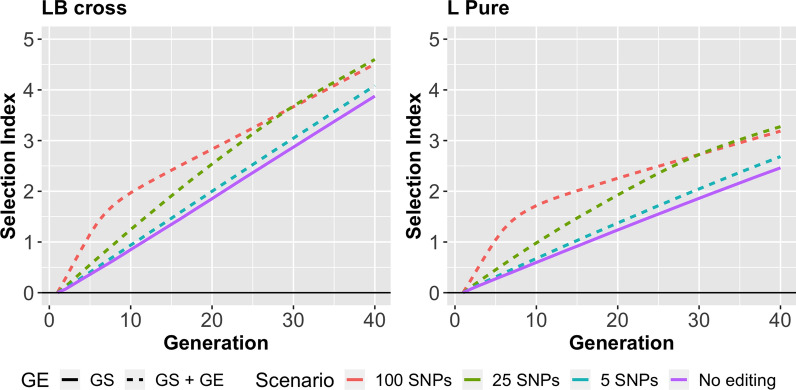
Genetic gain in the selection index for different numbers of edited SNPs. The dashed lines are the results from gene editing, which correspond from top to bottom to the editing of 100 SNPs, 25 SNPs and 5 SNPs, respectively. Generally, the L-B cross scenarios have a higher genetic gain. The more the SNPs are edited, the faster is the genetic gain in the first generations

Figure 3 shows the genetic gain in the selection index for different numbers of edits. Although the genetic gain increases with an increasing number of edits, it did not increase by more than 81% until generation 20.

In the L-B-cross scenarios, GE of five SNPs per edited animal brings 8% more genetic gain, editing of 25 SNPs provided 37% more genetic gain, and editing of 100 SNPs provided 53% more genetic gain until generation 20. In the L-Pure scenario, editing of five SNPs provided 11% more genetic gain, editing of 25 SNPs provided 56% more genetic gain, and editing of 100 SNPs provided 82% more genetic gain until generation 20. The numerical results are in Additional file 1: Table S4.

Gene editing was more beneficial for the L-Pure scenario because several QTL with an effect on meat production were not segregating in the layer line, so they had to be brought into the population by GE. This increased the genetic variance of the breeding line. After generation 20, GE no longer provided extra genetic gain because the QTL that were still segregating had only small effects.

### Overall health and the negative off-target effects

**Fig. 4:**
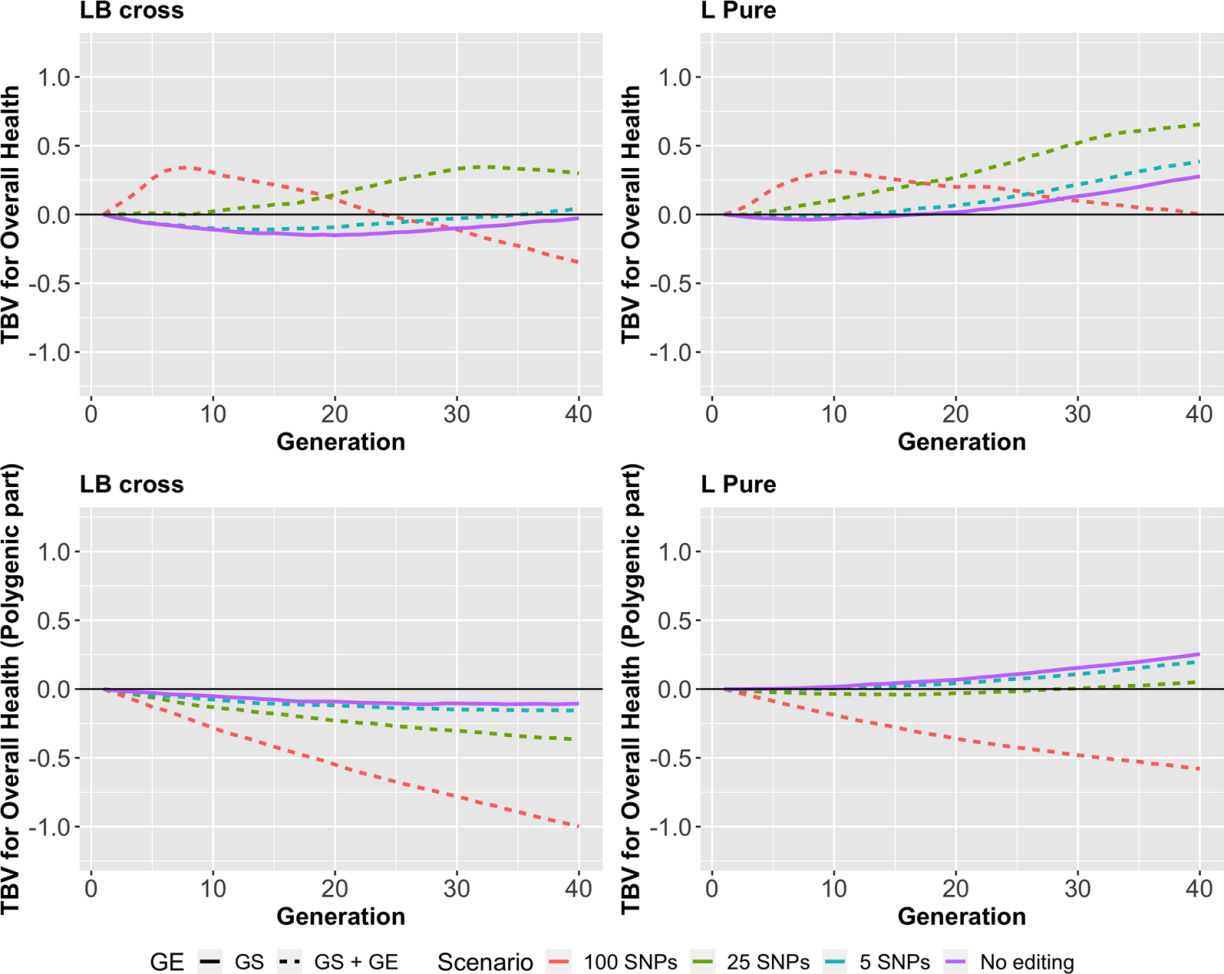
Changes in overall health over generations. Left: The L-B cross scenario. Right: The L-Pure scenario. Top: Breeding value for overall health. Bottom: Polygenic part of the breeding value for overall health. Although gene editing had a negative effect on the polygenic part of the TBV for overall health, the overall effect of gene editing on overall health was neutral when not more than 25 SNPs were edited

In this simulation, we assumed that off-target edits could have a negative effect on overall health. The effect of an off-target edit on the overall health of an animal was simulated by adding a negative value to the polygenic part of the breeding value for the overall health trait. Figure 4 (bottom) shows that the polygenic part of the TBV for overall health indeed decreases with an increasing number of edits. However, Fig. 4 (top) shows that editing of five SNPs or 25 SNPs increased the overall health slightly. This was because off-target edits with negative overall health effects were rare, and the edited SNPs had, on average, positive effects on overall health. Their effects on overall health were positive because the SNPs were chosen based on their estimated effects on the selection index, which placed a positive weight on the overall health trait. Editing of 100 SNPs per animal reduced the overall health after generation 10. The numerical results are in Additional file 1: Table S3.

### The change in genetic variances and heritabilities

**Fig. 5:**
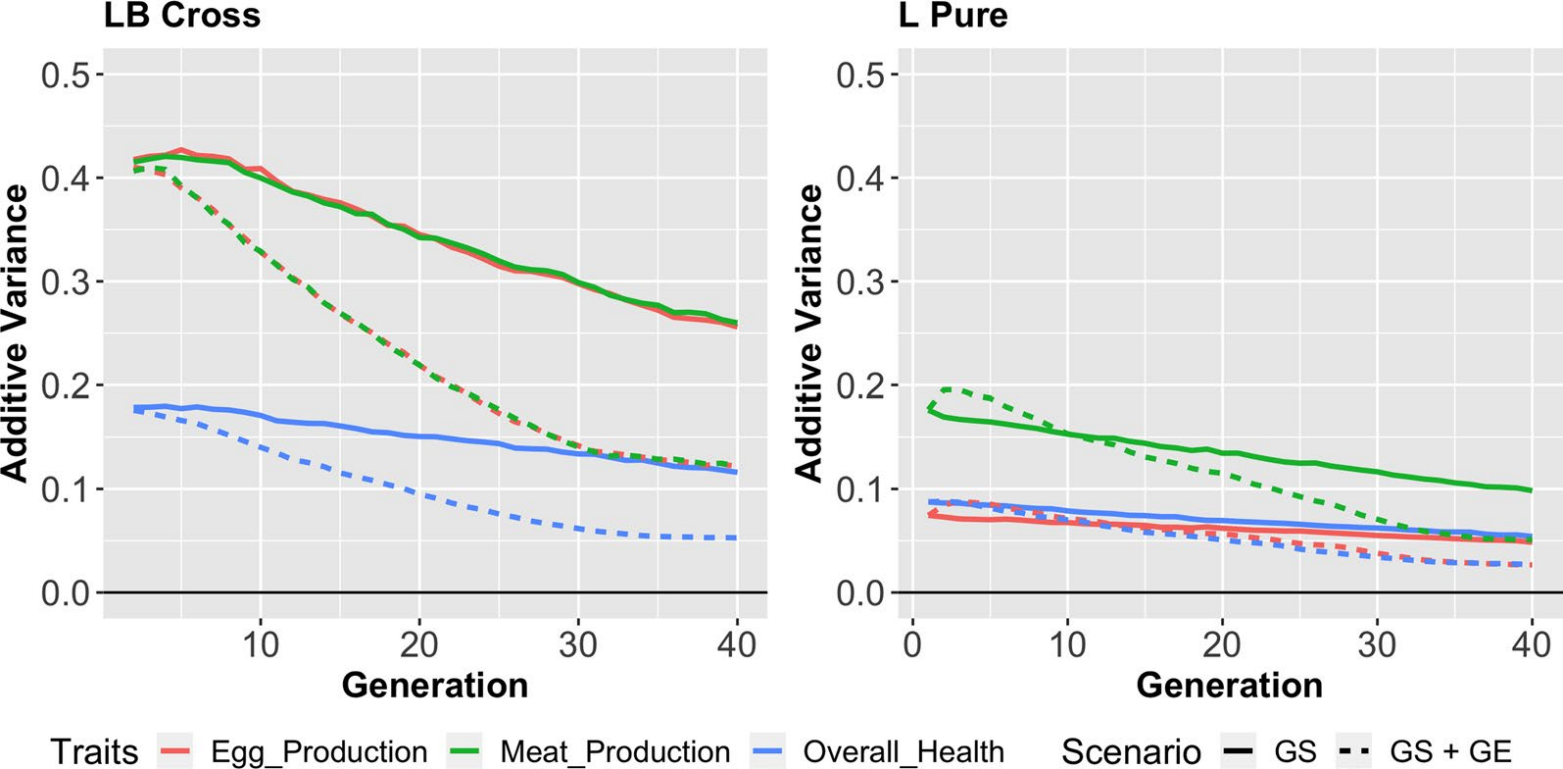
Change in additive variance when 25 SNPs could be edited for each of the 25 edited animals. Left: Overall change in the additive variance in the L-B cross. Right: Overall change in the additive variance in the L-Pure scenario. The slight rise in additive variance in the L-Pure scenario (right) with gene editing (dashed line) reflects that gene editing can bring back the variants in QTL that were removed from the population

The changes of the genetic variances over generations are shown in Fig. 5. Gene editing sped up the decrease of the additive variance by fixing the genotypes in the beneficial phase, which also reduced the heritabilities of the traits. The L-B cross scenario has larger additive variances for all traits because the line was derived from a cross between divergently selected lines. In the first generations, GE can bring back the variants of the QTL that were fixed, which could increase the additive variance. This explains the slight rise of the additive variance in the L-Pure scenario with GE. Generally, the more SNPs were edited per animal, the faster was the decrease in the genetic variances.

## Discussion

Two different breeding program designs for breeding dual-purpose chickens were compared with and without GE. The simulation protocol made some simplifying assumptions, e.g., that the QTL are not in LD or that editing male parents was possible, as discussed below. Thus, the simulation results allow only to draw general conclusions on the suitability of GE. Generally, GE combined with genomic selection results in greater genetic gain in breeding programs for dual-purpose chicken. The simulation focused on the relationship between the effects of on-target and off-target edits. Considering that off-target edits are generally rare when known QTL are edited [21, 22], the edits of falsely significant SNPs have, in fact, also to be considered as off-target edits. Consequently, the occurrence of off-target edits is not negligible in breeding programs for quantitative traits. The probability that off-target edits have a negative effect on the overall health is, however, unknown.

### The effects of off-target edits

Although the simulation assumed that GE caused negative effects on overall health due to off-target edits with a certain probability, the overall health of the population did not decrease beyond its initial value. This was because the overall health was included in the selection index. In reality, many overall health-related traits exist and, in practice, it might not be possible to record all of them and to include them in the selection index. In that case, it would not be possible to account for all effects of off-target edits by index selection.

The more frequently off-target edits occur during GE, the higher the index weight placed on overall health needs to be, which decreases the rate of genetic gain that can be achieved in a breeding program. For example, if the selection index is changed to (0.35, 0.35, 0.30), the genetic gain for egg production and meat production in the L-B-Cross + GE (editing 25 SNPs) scenario decreased by more than 12% in 20 generations. If the traits of interest are polygenic, the small positive effects of the edited SNPs on production traits may not compensate for the negative effects of off-target edits on overall health traits.

Gene editing, as opposed to artificial selection, does not cause a deterioration of genetic diversity in the vicinity of large QTL that are subject to selective sweeps. The local inbreeding caused by artificial selection can lead to inbreeding depression, which can be avoided by GE.

### The optimal number of SNPs to edit

In the simulation, we compared three numbers of SNPs that could be edited at the same time in a single animal. Although editing 100 SNPs in one go might not be possible with the current editing technologies, this extreme case was simulated for comparison. In previous studies [7], which did not consider the risk of off-target effects, it was proposed that editing the top sires would be the most beneficial. It was concluded that editing more quantitative trait nucleotides (QTN) would always bring more genetic gain. In the present study, we assumed a certain risk that editing an SNP has a negative effect on overall health. Consequently, the more SNPs are edited, the larger the index weight of the overall health trait should be in order to keep the overall health of the population stable. When many SNPs are edited, then many of them may have tiny effects on the selection index, although all have the same risk of causing off-target edits with negative effects on overall health. Thus, there should be an optimum for the number of SNPs to be edited in a single animal. In the current simulation, only small risks for negative effects on overall health were simulated. If the negative effects were larger than in the simulation, the scenarios editing 25 SNPs might also fail to be superior to GS alone. In addition, if 100 SNPs were edited per animal, then the genetic gain per generation decreased within less than 10 generations below the genetic gain that would be achieved without GE, which implies that GE should be discontinued after some generations.

### Choice of the index weights

The different selection indices reflect different breeding goals. In the simulation, the index weights were chosen so that the overall health was kept stable in the 100 generations of historic selection, and in the 40 subsequent generations for all scenarios that did not implement GE. In practice, different selection indices have been proposed for the production traits of dual-purpose chickens, reflecting different possibilities in the future chicken industry. According to a report issued by the chicken breeding company Lohmann, it is possible in the best-case scenario that meat chicken production accounts for half of the farmers’ income [23]. Therefore, we simply assumed that the index weights were equal for the two production traits.

### Scenario comparison

The L-B cross scenario provided a slightly larger genetic gain in the selection index than the L-Pure scenario, which was due to the higher genetic diversity in that line. Although the genetic gain in the L-B cross scenario was larger, the higher genetic diversity causes the production animals to be more heterogeneous, which could be considered a disadvantage by the farmers and consumers.

When looking at individual traits, the two scenarios differ strongly. The L-B cross scenario, which improves a synthetic line that was obtained from a Layer-Broiler cross, has intermediate values for both meat and egg production traits, whereas the L-Pure scenario, which aimed at improving a laying hen line for dual-purpose use, had superior egg performance but a moderately low but steadily increasing meat performance. Gene editing in the L-B cross scenario improved both performance traits, while GE in the L-Pure scenario improved meat production, but reduced egg production. This was because the traits were negatively correlated. Quantitative trait loci with large effects on the selection index that were not yet fixed in the layer line tended to have opposite effects on meat production and egg production.

### Assumptions underlying the simulations

In our simulation, linkage was not simulated explicitly. The simulation accounted for linkage by assuming that the effect estimates of the QTL did not converge towards the true effects but towards suggestive effects that deviated slightly from the true effects.

In the current simulation, it is assumed that the negative correlations between traits are mainly caused by pleiotropic QTL. However, negative correlated traits could also be a consequence of linkage between closely-located QTL [24]. It has been proposed that GE could help to reduce the linkage [25]. In addition, the simulated traits were purely additive. In real populations, epistatic variance and dominance variance could be converted into additive variance in the course of many generations, which could cause the additive variance to decrease more slowly.

In the simulation protocol, it was assumed that animals could be edited after their genetic values have been calculated, which is, however, currently not possible. In mammals, one could edit cells derived from the respective males and produce edited clones by somatic cell nuclear transfer. In birds, GE is accomplished by editing of the embryo primordial germ cells. If the cells could be genotyped before or alongside GE, then it would be possible to hatch edited males that are genetically superior. Otherwise, the simulation results are likely to overestimate the potential of GE. One could also take advantage of the sire dam surrogate mating [26] which would allow to produce multiple male individuals possessing identical primordial germ cells. This would additionally promote genetic progress. Overall, it seems, however, justified to make the assumptions made in this study, having in mind that the results can be seen as the upper bound of what can be currently achieved.

In the simulation program, a single dual-purpose line was established. However, it might be argued that most commercial lines are either two-way or four-way hybrids. The reason is that the hybrid animals can utilize heterosis effects, which were not included in the simulation. In order to account for heterosis effects in practice, it might be appropriate to establish two dual-purpose lines and to sell their crosses as production animals. The simulation could thus be seen as half of a hybrid breeding program.

Negatively correlated production traits also exist in other livestock. For example, it could be desired to combine milk production and meat production in cattle. Our simulation, although discussed in the context of chicken breeding, could also provide insights on the breeding of other species.

### Public concerns and ethical reflection

Even though additional genetic gain was achieved by GE in our simulation study, this result cannot been considered independently from ethical and societal concerns related to GE. First, consumers’ attitude toward GE is relevant for reasons of practical feasibility. It is well discussed that consumers would pay more for not genetically modified (Non-GMO) products [27, 28]. In most cases, GE is still considered as GMO and are not preferred [29]. Considering that GE is beneficial only as long as alleles with reasonable effect sizes have not been fixed in the population, GE may not be appealing enough for the farmers. Second, the ethical concerns related to GE, such as on animal welfare, instrumentalization of animals and justice need to be taken seriously- as part of responsible innovation [30–32]. This reflection should not only focus on new ethical issues, but also on existing issues that will be ameliorated, perpetuated or aggravated by GE [33]. From this perspective, the indication that GE may accelerate genetic progress in dual-purpose chicken is not a direct argument to justify the application of GE, but nonetheless it is a relevant consideration in the ethical assessment of both GE and how to best address the problem of the killing of the male layer chicks direct after hatching. As a result breeders should take these wider ethical and societal issues seriously before any implementation into the breeding programs.

## Conclusions

Genomic selection breeding programs with and without the implementation of GE were simulated. Our simulation demonstrated a general increase in genetic gain when genomic selection is used together with GE. Gene editing is helpful in achieving extra genetic gain by changing the allele frequencies of segregating alleles, and by introducing new alleles into the population. The more alleles are edited per animal, the smaller is their average effect on the selection index. Nevertheless, each edit has the same probability of causing off-target edits with a negative effect on overall health. Therefore, an optimum number of edits per animal should exist. The impact of negative off-target effects, when assumed to be mild, could be balanced by placing an appropriate weight on the overall health in the selection index. The overall benefit of GE erased after some generations because the large-effect alleles became fixed. Hence, GE could be beneficial only when alleles with reasonable effect sizes are segregating and detectable. When the consumer preference and the price difference between the genome-edited chicken and the other chickens were to be considered, the findings from this study might not be sufficient to recommend the use of GE in breeding programs for quantitative traits.

### Supplementary Information

**Additional file 1: Table S1.** Changes in average true genetic values (TGV) for egg production in different scenarios and different lines. The change in true genetic values for egg production (until 1, 10, 20, and 40 generations) compared to the -100 generation. **Table S2.** Changes in average true genetic values (TGV) for meat production in different scenarios and different lines. The change in true genetic values for meat production (until 1, 10, 20, and 40 generations) compared to the -100 generation. **Table S3.** Changes in average true genetic values (TGV) for overall health in different scenarios and different lines. The change in true genetic values for overall health (until 1, 10, 20, and 40 generations) compared to the -100 generation. **Table S4.** Changes in average true genetic values (TGV) changes of (dual-purpose) trait index in different scenarios and different lines. The change in true genetic values for the (dual-purpose) trait index (until 1, 10, 20, and 40 generations) compared to the -100 generation.

**Additional file 2.** Proofs for the conditional distributions of EBV and for the variance of the estimate of an SNP-effect.

## Data Availability

The simulation results are in the additional files.
