# Supplementary material for: Simulation of dual-purpose chicken breeding programs implementing gene editing
Source: Genet Sel Evol. 2024 Jan 17;56:7. doi: 10.1186/s12711-023-00874-3 (PMC10795215; doi:10.1186/s12711-023-00874-3)
Supplement: Supplementary file 1 — Additional file 1: Table S1. Changes in average true genetic values (TGV) for egg production in different scenarios and different lines. The change in true genetic values for egg production (until 1, 10, 20, and 40 generations) compared to the -100 generation. Table S2. Changes in average true genetic values (TGV) for meat production in different scenarios and different lines. The change in true genetic values for meat production (until 1, 10, 20, and 40 generations) compared to the -100 generation. Table S3. Changes in average true genetic values (TGV) for overall health in different scenarios and different lines. The change in true genetic values for overall health (until 1, 10, 20, and 40 generations) compared to the -100 generation. Table S4. Changes in average true genetic values (TGV) changes of (dual-purpose) trait index in different scenarios and different lines. The change in true genetic values for the (dual-purpose) trait index (until 1, 10, 20, and 40 generations) compared to the -100 generation. [file 12711_2023_874_MOESM1_ESM.docx]

Additional file:

Results with different number of edits

**Table S1 Average true genetic value (TGV) changes of egg count in different scenarios and different lines**

|  | **LB cross** | | | | **L Pure** | | | | |
| --- | --- | --- | --- | --- | --- | --- | --- | --- | --- |
|  | **TGV GE**  **100 edits** | **TGV GE**  **25 edits** | **TGV GE**  **5 edits** | **without GE** | | **TGV GE**  **100 edits** | **TGV GE**  **25 edits** | **TGV GE**  **5 edits** | **without GE** |
| Generation 1 | 8.83  (1.82) | 8.83  (1.83) | 8.83  (1.83) | 8.83  (1.83) | | 19.80  (2.00) | 19.80  (2.00) | 19.80  (2.00) | 19.80  (2.00) |
| Generation 10 | 11.41  (2.03) | 10.53  (1.91) | 10.16  (1.86) | 10.00  (1.86) | | 14.71  (2.01) | 18.17 (1.92) | 19.47 (1.99) | 19.83  (1.99) |
| Generation 20 | 12.60  (2.06) | 12.27  (1.93) | 11.61  (1.94) | 11.41  (1.90) | | 14.84  (2.07) | 16.68  (1.98) | 19.19  (2.00) | 19.82 (2.00) |
| Generation 30 | 13.83  (2.06) | 13.72  (1.96) | 13.00  (2.00) | 12.74  (1.93) | | 15.11  (2.07) | 15.42  (2.03) | 18.89  (2.00) | 19.76 (2.01) |
| Generation 40 | 15.03  (2.10) | 14.95  (2.02) | 14.38  (2.04) | 14.08 (1.98) | | 15.37  (2.08) | 15.34  (2.08) | 18.56  (2.01) | 19.73 (2.02) |

Standard deviation over all 100 replicates are indicated in brackets

**Table S2 Average true genetic value (TGV) changes of daily gain in different scenarios and different lines**

|  | **LB cross** | | | | **L Pure** | | | | |
| --- | --- | --- | --- | --- | --- | --- | --- | --- | --- |
|  | **TGV GE**  **100 edits** | **TGV GE**  **25 edits** | **TGV GE**  **5 edits** | **without GE** | | **TGV GE**  **100 edits** | **TGV GE**  **25 edits** | **TGV GE**  **5 edits** | **without GE** |
| Generation 1 | 8.98  (1.82) | 8.98  (1.82) | 8.98  (1.82) | 8.98  (1.82) | | -1.99  (2.00) | -1.99  (2.00) | -1.99  (2.00) | -1.99  (2.00) |
| Generation 10 | 11.45  (2.05) | 10.59  (1.92) | 10.21  (1.87) | 10.15  (1.85) | | 7.46  (1.96) | 2.18  (1.96) | 0.14  (2.03) | -0.42  (2.03) |
| Generation 20 | 12.69  (2.05) | 12.21  (1.99) | 11.59  (1.94) | 11.45  (1.89) | | 8.85  (2.02) | 6.08  (2.09) | 2.23  (2.06) | 1.27  (2.06) |
| Generation 30 | 13.83  (2.08) | 13.68  (2.10) | 12.95  (1.98) | 12.80  (1.95) | | 9.89  (2.08) | 9.30  (2.15) | 4.22  (2.06) | 2.91  (2.09) |
| Generation 40 | 15.03  (2.18) | 14.92  (2.15) | 14.28  (2.03) | 14.09  (2.00) | | 10.93  (2.16) | 10.76  (2.16) | 6.14  (2.17) | 4.45  (2.13) |

Standard deviation over all 100 replicates are indicated in brackets

**Table S3 Average true genetic value (TGV) changes of fitness in different scenarios and different lines**

|  | **LB cross** | | | | **L Pure** | | | | |
| --- | --- | --- | --- | --- | --- | --- | --- | --- | --- |
|  | **TGV GE**  **100 edits** | **TGV GE**  **25 edits** | **TGV GE**  **5 edits** | **without GE** | | **TGV GE**  **100 edits** | **TGV GE**  **25 edits** | **TGV GE**  **5 edits** | **without GE** |
| Generation 1 | -0.16  (1.18) | -0.16  (1.18) | -0.16  (1.18) | -0.16  (1.18) | | -0.06  (1.31) | -0.06 (1.31) | -0.06  (1.31) | -0.06  (1.31) |
| Generation 10 | 0.14  (1.33) | -0.14  (1.26) | -0.26  (1.21) | -0.27  (1.20) | | 0.26  (1.31) | 0.05  (1.34) | -0.07  (1.30) | -0.09 (1.32) |
| Generation 20 | -0.05  (1.36) | -0.02  (1.33) | -0.26  (1.23) | -0.31  (1.24) | | 0.14  (1.34) | 0.21  (1.34) | 0.01  (1.32) | -0.04  (1.34) |
| Generation 30 | 0.27  (1.35) | 0.17  (1.35) | -0.19  (1.26) | -0.26  (1.23) | | 0.04  (1.36) | 0.46  (1.33) | 0.16  (1.32) | 0.08  (1.36) |
| Generation 40 | -0.51  (1.35) | 0.14  (1.38) | -0.11  (1.27) | -0.19  (1.26) | | -0.05  (1.37) | 0.60  (1.33) | 0.33  (1.34) | 0.22  (1.37) |

Standard deviation over all 100 replicates are indicated in brackets

**Table S4 Average true genetic value (TGV) changes of (dual-purpose) trait index in different scenarios and different lines**

|  | **LB cross** | | | | **L Pure** | | | | |
| --- | --- | --- | --- | --- | --- | --- | --- | --- | --- |
|  | **TGV GE**  **100 edits** | **TGV GE**  **25 edits** | **TGV GE**  **5 edits** | **without GE** | | **TGV GE**  **100 edits** | **TGV GE**  **25 edits** | **TGV GE**  **5 edits** | **without GE** |
| Generation 1 | 6.64  (0.84) | 6.64  (0.84) | 6.64  (0.84) | 6.64  (0.84) | | 6.66  (0.84) | 6.66  (0.84) | 6.66  (0.84) | 6.66  (0.84) |
| Generation 10 | 8.61  (0.87) | 7.88  (0.86) | 7.58  (0.84) | 7.49  (0.84) | | 8.38  (0.85) | 7.64  (0.85) | 7.34  (0.86) | 7.26  (0.84) |
| Generation 20 | 9.47  (0.86) | 9.18  (0.87) | 8.64  (0.85) | 8.49  (0.85) | | 8.92  (0.87) | 8.59  (0.87) | 8.03  (0.87) | 7.90  (0.84) |
| Generation 30 | 10.31  (0.86) | 10.32  (0.87) | 9.68  (0.84) | 9.51  (0.85) | | 9.39  (0.88) | 9.39  (0.89) | 8.71  (0.87) | 8.52  (0.85) |
| Generation 40 | 11.15  (0.86s) | 11.24  (0.87) | 10.72  (0.85) | 10.51  (0.85) | | 9.85  (0.89) | 9.94  (0.88) | 9.34  (0.88) | 9.12  (0.86) |

Standard deviation over all 100 replicates are indicated in brackets
