# Supplementary material for: Simulation of dual-purpose chicken breeding programs implementing gene editing
Source: Genet Sel Evol. 2024 Jan 17;56:7. doi: 10.1186/s12711-023-00874-3 (PMC10795215; doi:10.1186/s12711-023-00874-3)
Supplement: Supplementary file 2 — Additional file 2. Proofs for the conditional distributions of EBV and for the variance of the estimate of an SNP-effect. [file 12711_2023_874_MOESM2_ESM.docx]

**Appendix A: Conditional distribution of EBVs**

Let $u$ denote the true breeding value of an animal, and let $\hat{u}$ denote its estimated breeding value, which are both normally distributed with mean 0. We assume that $cov\left( u,\hat{u} \right)=var\left( \hat{u} \right)$. The correlation between true and estimated breeding value equals

$$r=\frac{cov\left( u,\hat{u} \right)}{\sigma_{u}\sigma_{\hat{u}}}=\frac{\sigma_{\hat{u}}^{2}}{\sigma_{u}\sigma_{\hat{u}}}=\frac{\sigma_{\hat{u}}}{\sigma_{u}},$$

where $\sigma_{\hat{u}}^{2}=\mathrm{var}\left( \hat{u} \right)$, and $\sigma_{u}^{2}=\mathrm{var}\left( u \right)$. The 2-vector $\left( u,\hat{u} \right)$ has a bivariate normal distribution. From the properties of jointly normal random variables, it follows that the conditional distribution of $\hat{u}$ given $u$ is normal with mean

$$E\left( \hat{u}|u \right)=E\left( \hat{u} \right)+r\frac{\sigma_{\hat{u}}}{\sigma_{u}}\left( u-E\left( u \right) \right)=r^{2}u,$$

and variance

$$\mathrm{var}\left( \hat{u}|u \right)=\left( 1-r^{2} \right)\sigma_{\hat{u}}^{2}=\left( 1-r^{2} \right)r^{2}\sigma_{u}^{2}.$$

**Appendix B: Variance of an SNP-effect estimate**

We consider a biallelic SNP with alleles 0 and 1. Let $y$ denote the vector with observations, and let $\bar{y}_{00}$, $\bar{y}_{01},$ and $\bar{y}_{11}$ be the average trait values of animals with genotypes 00, 01, and 11, respectively. We consider the simple estimator

$$\hat{a}=\left( 1-2p \right)\bar{y}_{01}+p\bar{y}_{11} - \left( 1-p \right)\bar{y}_{00}$$

for the additive effect $a$ of the SNP, where $p$ is the allele frequency of allele 1. The estimator is unbiased for the linear regression model $y_{i}=\mu+x_{i}a+e_{i}$, where $x_{i}$ is the allele content of animal $i$. That is,

$$E\left( \hat{a} \right)=\left( 1-2p \right)\left( \mu+a \right)+p\left( \mu+2a \right)-\left( 1-p \right)\mu=a.$$

For a population in Hardy-Weinberg equilibrium, the variance of the estimator is

$$Var\left( \hat{a} \right)=Var\left( \left( 1-2p \right)\bar{y}_{01}+p\bar{y}_{11} - \left( 1-p \right)\bar{y}_{00} \right)$$

$$=\left( 1-2p \right)^{2}Var\left( \bar{y}_{01} \right)+p^{2}Var\left( \bar{y}_{11} \right)+\left( 1-p \right)^{2}Var\left( \bar{y}_{00} \right)$$

$$\approx\left( 1-2p \right)^{2}\frac{V_{P}}{2p\left( 1-p \right)N}+p^{2}\frac{V_{P}}{p^{2}N}+\left( 1-p \right)^{2}\frac{V_{P}}{\left( 1-p \right)^{2}N}$$

$$=\frac{V_{P}}{N}\left[ 2+\frac{\left( 1-2p \right)^{2}}{2p\left( 1-p \right)} \right]$$

$$=\frac{V_{P}}{N}\left[ \frac{{4p\left( 1-p \right)+\left( 1-2p \right)}^{2}}{2p\left( 1-p \right)} \right]$$

$$=\frac{V_{P}}{N}\frac{1}{2p\left( 1-p \right)}.$$

This estimator was only considered because its variance could easily be computed. In practice, other estimators are preferred.
